# Supplementary material for: One-step construction of circularized nanodiscs using SpyCatcher-SpyTag
Source: Nat Commun. 2021 Sep 14;12:5451. doi: 10.1038/s41467-021-25737-7 (PMC8440770; doi:10.1038/s41467-021-25737-7)
Supplement: Supplementary file 1 — Supplementary Information [file 41467_2021_25737_MOESM1_ESM.pdf]

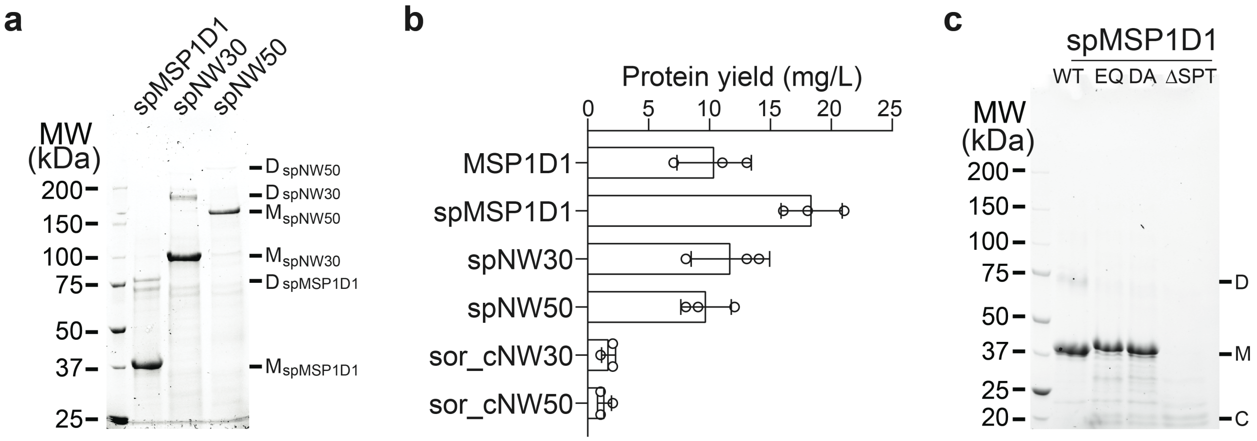

Supplementary Figure 1. Characterization of MSPs circularized via SpyCatcher-SpyTag. **a,** Representative SDS-PAGE of proteins purified from cells induced with 1 mM IPTG at 37 °C. As compared to Fig. 1B, more oligomers were formed. n = 3 independent experiments. **b**, Protein yields of MSPs. sor_cNW30 and sor_cNW50 stand for the circularized NW30 and NW50 using the sortase-mediated ligation approach. Data are shown as mean ± s.d., n = 3 independent experiments. **c,** Representative SDS-PAGE of spMSP1D1 in comparison with the indicated mutants. ΔSPT, spMSP1D1 lacking the SpyTag. M, monomer; D, dimer; C, cleaved fragments. n = 3 independent experiments.

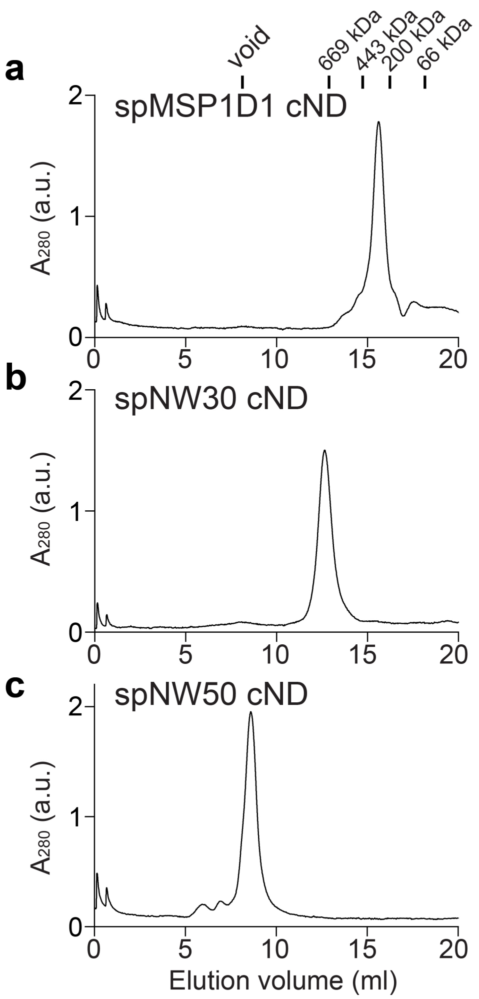

Supplementary Figure 2. SEC profiles of purified cNDs.
a-c, Purified cNDs from Fig. 2 were analyzed by a second SEC to evaluate their homogeneity. These cNDs were reconstituted using spMSP1D1 (a), spNW30 (b), spNW50 (c). a. u., arbitrary units.

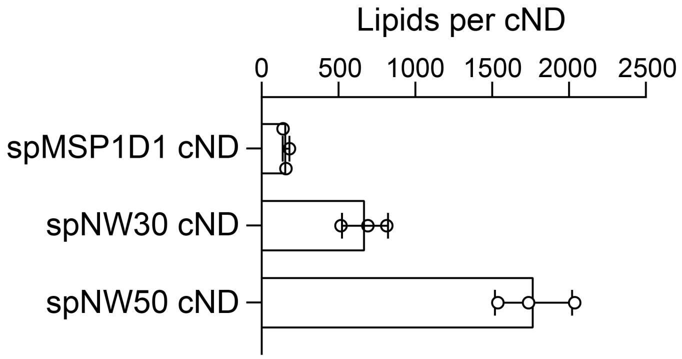

Supplementary Figure 3. Quantification of lipids per nanodisc.
cNDs were prepared using PC lipids doped with 0.5% Rhodamine-PE. The lipid/protein ratios were calculated by determining protein concentrations via densitometry and lipid concentrations via the fluorescence emission of Rhodamine-PE. Data are shown as mean ± s.d., n = 3 independent experiments.

a

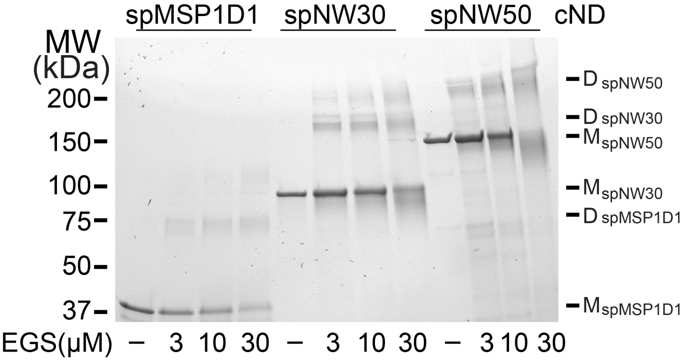

b

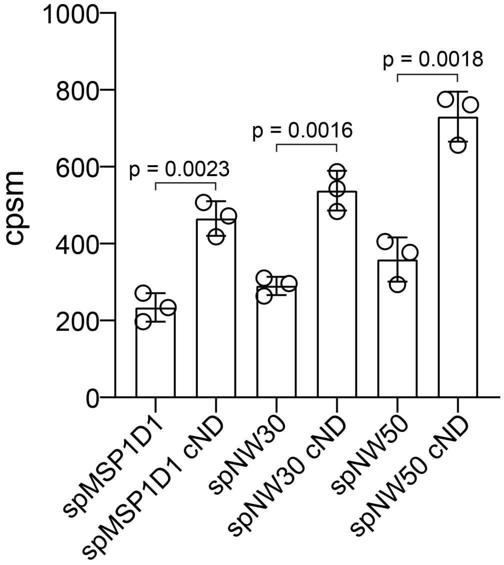

Supplementary Figure 4. cNDs were formed by two copies of spMSPs.
a, Representative SDS-PAGE of the indicated cNDs that were subjected to crosslinking experiments using an increasing amount of EGS. M, monomer; D, dimer. n = 3 independent experiments. **b**, The molecular brightness of spMSPs and cNDs labeled with FITC were determined by fluorescence fluctuation spectroscopy. cpsm, counts per second per molecule. Statistics were determined using two-tailed t-test. Data are shown as mean ± s.d., n = 3 independent experiments.

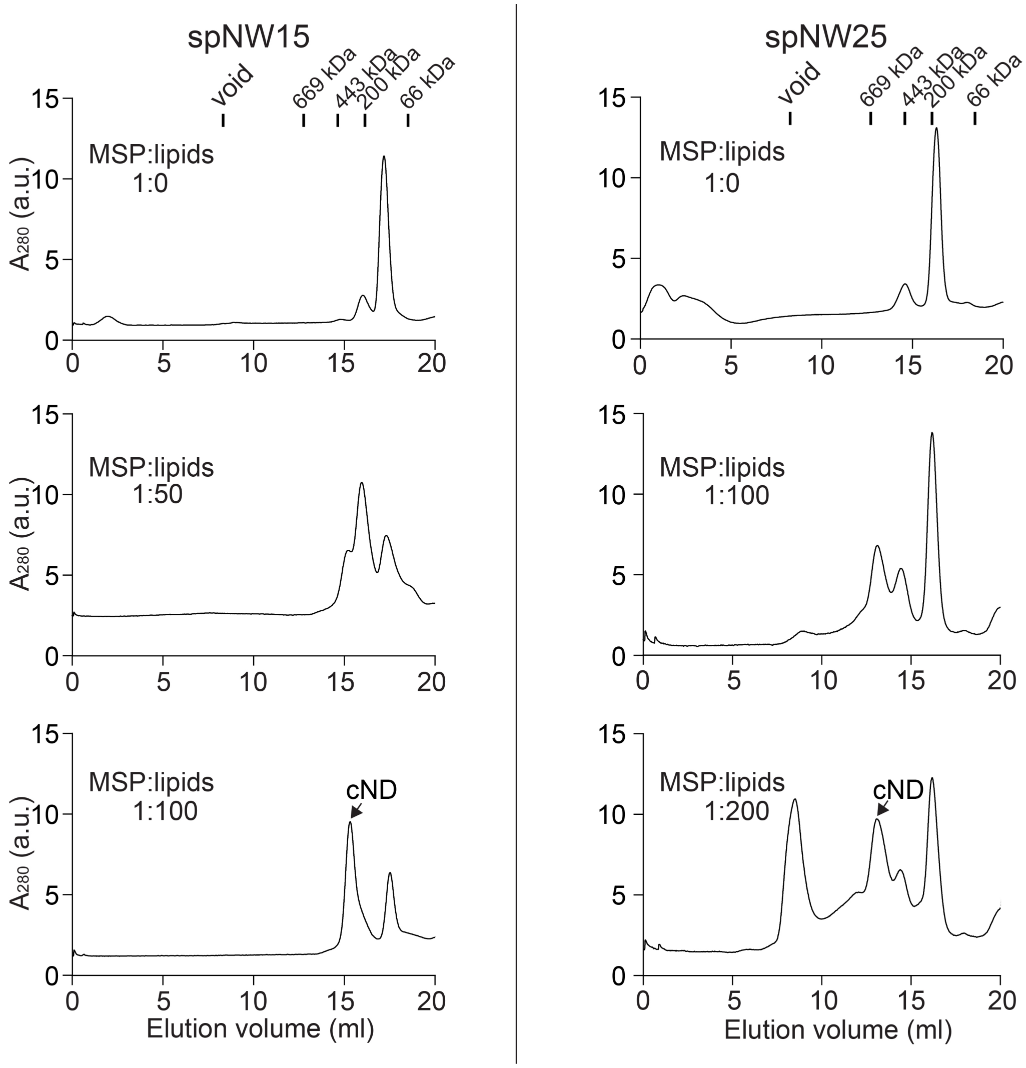

Supplementary Figure 5. SEC profile of spNW15 and spNW25 cNDs.
cNDs were reconstituted at the indicated protein/lipid ratios and subjected to SEC using a Superose 6 10/300 GL column. a. u., arbitrary units.

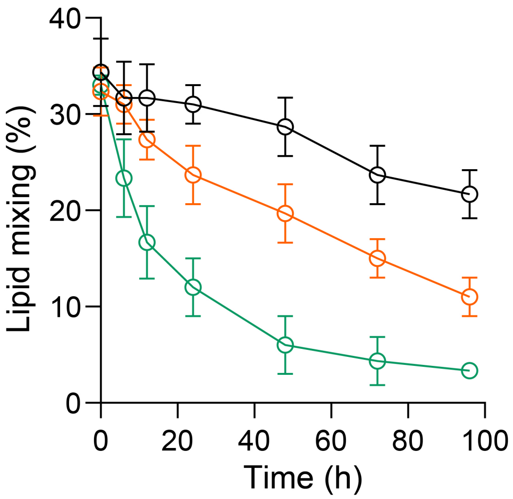

Supplementary Figure 6. Stability of cNDs in comparison with nanodiscs and liposomes. v-SNAREs were reconstituted in spMSP1D1 cNDs (black), non-circularized MSP1D1 nanodiscs (orange), and liposomes (green). Samples were kept at 4 °C for the indicated time and then subjected to the lipid-mixing fusion assay with liposomes bearing t-SNAREs. The lipid-mixing efficiencies were plotted as a function of the time of the samples kept at 4 °C. Data are shown as mean ± s.d., n = 3 independent experiments.

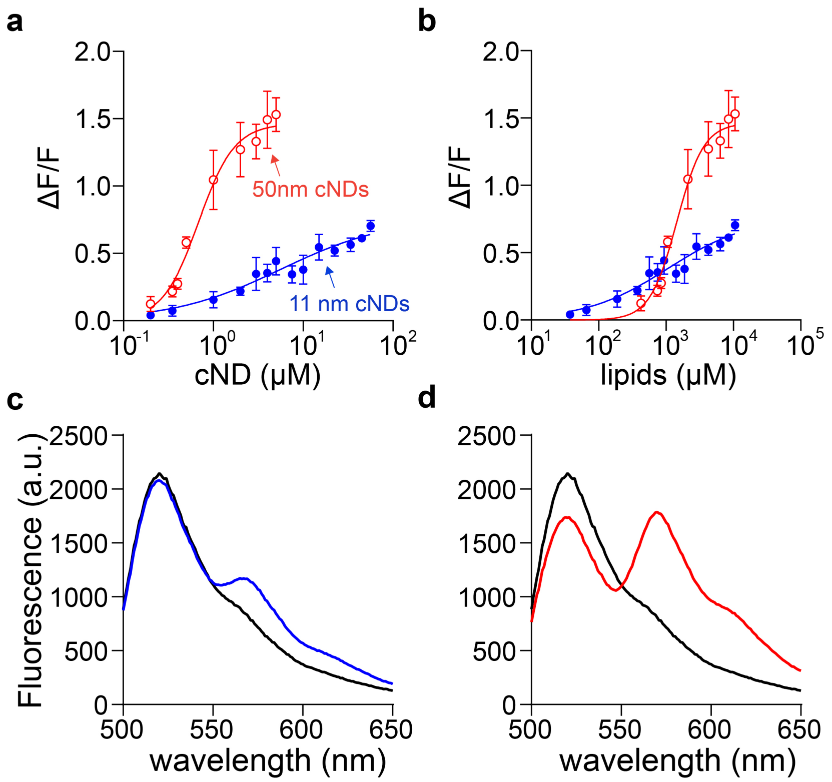

Supplementary Figure 7. Characterization of syt1 and cpx2 binding to cNDs.
a-b, Binding of syt1 to cNDs was monitored using fluorescence spectrometry, and the ΔF/F values were plotted as a function of cND (a) or lipid (b) concentrations. The data were fitted to a one-site binding equation with a hill coefficient using GraphPad. **c-d**, Fluorescence emission spectrum of OG-labelled cpx2 (10 nM) in the absence (black) or presence of DiI-labelled 11 (c, blue) and 50 (d, red) nm cNDs (10 μM). Throughout figure, data are shown as mean ± s.d., n = 3 independent experiments. a. u., arbitrary units.

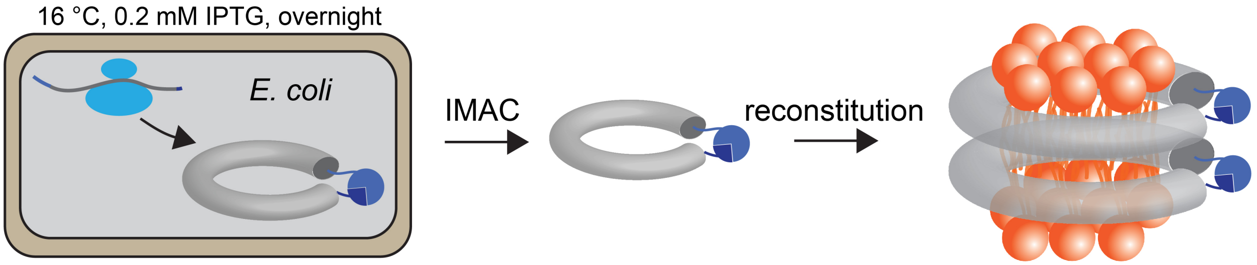

Supplementary Figure 8. Workflow of using spMSPs for cND reconstitution.
Owing to the high yield and circularization efficiency, circularized spMSPs were purified via a single step using immobilized-metal affinity chromatography (IMAC). The purified proteins were then used for the reconstitution of cNDs that could be readily isolated from SEC with great monodispersity. SpyCatcher, sapphire blue; SpyTag, navy blue.

Supplementary Table I. Protein sequence of spMSPs

spMSP1D1	MGSSHHHHHHSSGLVPRGSHMASMTGGQQMGRGSGAMVTTLSGLSGEQG<br>PSGDMTTEEDSATHIKFSKRDEDGRELAGATMELRDSSGKTISTWISDGHVKD<br>FYLYPGKYTFVETAAPDGYEVATAITFTVNEQGQVTVNGEATKGDAHTSTFSK<br>LREQLGPVTQEFWDNLEKETEGLRQEMSKDLEEVKAKVQPYLDDFQKKWQE<br>EMELYRQKVEPLRAELQEGARQKLHELQEKLSPLGEEMRDRARAHVDALRTH<br>LAPYSDELRQRLAARLEALKENGGARLAEYHAKATEHLSTLSEKAKPALEDLR<br>QGLLPVLESFKVSFLSALEEYTKKLNTQVPTIVMVDAYKRYK
spNW15	MGSSHHHHHHSSGLVPRGSHMASMTGGQQMGRGSGAMVTTLSGLSGEQG<br>PSGDMTTEEDSATHIKFSKRDEDGRELAGATMELRDSSGKTISTWISDGHVKD<br>FYLYPGKYTFVETAAPDGYEVATAITFTVNEQGQVTVNGEATKGDAHTSTFSK<br>LREQLGPVTQEFWDNLEKETEGLRQEMSKDLEEVKAKVQPYLDDFQKKWQE<br>EMELYRQKVEPLRAELQEGARQKLHELQEKLSPLGEEMRDRARAHVDALRTH<br>LAPYSDELRQRLAARLEALKENGGARLAEYHAKATEHLSTLSEKAKPALEDLR<br>QGLLPVLESFKVSFLSALEEYTKKLNTQLPGTGAAALEVPTIVMVDAYKRYK
spNW25	MGSSHHHHHHSSGLVPRGSHMASMTGGQQMGRGSGAMVTTLSGLSGEQG<br>PSGDMTTEEDSATHIKFSKRDEDGRELAGATMELRDSSGKTISTWISDGHVKD<br>FYLYPGKYTFVETAAPDGYEVATAITFTVNEQGQVTVNGEATKGDAHTSTFSK<br>LREQLGPVTQEFWDNLEKETEGLRQEMSKDLEEVKAKVQPYLDDFQKKWQE<br>EMELYRQKVEPLRAELQEGARQKLHELQEKLSPLGEEMRDRARAHVDALRTH<br>LAPYSDELRQRLAARLEALKENGGARLAEYHAKATEHLSTLSEKAKPALEDLR<br>QGLLPVLESFKVSFLSALEEYTKKLNTQGTPVTQEFWDNLEKETEGLRQEMS<br>KDLEEVKAKVQPYLDDFQKKWQEEMELYRQKVEPLRAELQEGARQKLHELQ<br>EKLSPLGEEMRDRARAHVDALRTHLAPYSDELRQRLAARLEALKENGGARLA<br>EYHAKATEHLSTLSEKAKPALEDLRQGLLPVLESFKVSFLSALEEYTKKLNTQL<br>PGTGAAALEVPTIVMVDAYKRYK
spNW30	MGSSHHHHHHSSGLVPRGSHMASMTGGQQMGRGSGAMVTTLSGLSGEQG<br>PSGDMTTEEDSATHIKFSKRDEDGRELAGATMELRDSSGKTISTWISDGHVKD<br>FYLYPGKYTFVETAAPDGYEVATAITFTVNEQGQVTVNGEATKGDAHTSTFSK<br>LREQLGVPVTQEFWDNLEKETEGLRQEMSKDLEEVKAKVQPYLDDFQKKWQ<br>EEMELYRQKVEPLRAELQEGARQKLHELQEKLSPLGEEMRDRARAHVDALRT<br>HLAPYSDELRQRLAARLEALKENGGARLAEYHAKATEHLSTLSEKAKPALEDL<br>RQGLLPVLESFKVSFLSALEEYTKKLNTQGTPVTQEFWDNLEKETEGLRQEM<br>SKDLEEVKAKVQPYLDDFQKKWQEEMELYRQKVEPLRAELQEGARQKLHEL<br>QEKLSPLGEEMRDRARAHVDALRTHLAPYSDELRQRLAARLEALKENGGARL<br>AEYHAKATEHLSTLSEKAKPALEDLRQGLLPVLESFKVSFLSALEEYTKKLNTQ<br>GTPVTQEFWDNLEKETEGLRQEMSKDLEEVKAKVQPYLDDFQKKWQEEMEL<br>YRQKVEPLRAELQEGARQKLHELQEKLSPLGEEMRDRARAHVDALRTHLAPY<br>SDELRQRLAARLEALKENGGARLAEYHAKATEHLSTLSEKAKPALEDLRQGLL<br>PVLESFKVSFLSALEEYTKKLNTQLPGTGAAALEVPTIVMVDAYKRYK
spNW50	MGSSHHHHHHSSGLVPRGSHMASMTGGQQMGRGSGAMVTTLSGLSGEQG<br>PSGDMTTEEDSATHIKFSKRDEDGRELAGATMELRDSSGKTISTWISDGHVKD<br>FYLYPGKYTFVETAAPDGYEVATAITFTVNEQGQVTVNGEATKGDAHTSTFSK<br>LREQLGPVTQEFWDNLEKETEGLRQEMSKDLEEVKAKVQPYLDDFQKKWQE<br>EMELYRQKVEPLRAELQEGARQKLHELQEKLSPLGEEMRDRARAHVDALRTH<br>LAPYSDELRQRLAARLEALKENGGARLAEYHAKATEHLSTLSEKAKPALEDLR<br>QGLLPVLESFKVSFLSALEEYTKKLNTQGTPVTQEFWDNLEKETEGLRQEMS<br>KDLEEVKAKVQPYLDDFQKKWQEEMELYRQKVEPLRAELQEGARQKLHELQ

	EKLSPLGEEMRDRARAHVDALRTHLAPYSDELRQRLAARLEALKENGGARLA<br>EYHAKATEHLSTLSEKAKPALEDLRQGLLPVLESFKVSFLSALEEYTKKLNTQG<br>TPVTQEFWDNLEKETEGLRQEMSKDLEEVKAKVQPYLDDFQKKWQEEMELY<br>RQKVEPLRAELQEGARQKLHELQEKLSPLGEEMRDRARAHVDALRTHLAPYS<br>DELRQRLAARLEALKENGGARLAEYHAKATEHLSTLSEKAKPALEDLRQGLLP<br>VLESFKVSFLSALEEYTKKLNTQGTPVTQEFWDNLEKETEGLRQEMSKDLEEV<br>KAKVQPYLDDFQKKWQEEMELYRQKVEPLRAELQEGARQKLHELQEKLSPL<br>GEEMRDRARAHVDALRTHLAPYSDELRQRLAARLEALKENGGARLAEYHAKA<br>TEHLSTLSEKAKPALEDLRQGLLPVLESFKVSFLSALEEYTKKLNTQGTPVTQE<br>FWDNLEKETEGLRQEMSKDLEEVKAKVQPYLDDFQKKWQEEMELYRQKVEP<br>LRAELQEGARQKLHELQEKLSPLGEEMRDRARAHVDALRTHLAPYSDELRQR<br>LAARLEALKENGGARLAEYHAKATEHLSTLSEKAKPALEDLRQGLLPVLESFK<br>VSFLSALEEYTKKLNTQLPGTGAAALEVPTIVMVDAYKRYK
spNW80	MGSSHHHHHHSSGLVPRGSHMASMTGGQQMGRGSGAMVTTLSGLSGEQG<br>PSGDMTTEEDSATHIKFSKRDEDGRELAGATMELRDSSGKTISTWISDGHVKD<br>FYLYPGKYTFVETAAPDGYEVATAITFTVNEQGQVTVNGEATKGDAHTSTFSK<br>LREQLGPVTQEFWDNLEKETEGLRQEMSKDLEEVKAKVQPYLDDFQKKWQE<br>EMELYRQKVEPLRAELQEGARQKLHELQEKLSPLGEEMRDRARAHVDALRTH<br>LAPYSDELRQRLAARLEALKENGGARLAEYHAKATEHLSTLSEKAKPALEDLR<br>QGLLPVLESFKVSFLSALEEYTKKLNTQGTPVTQEFWDNLEKETEGLRQEMS<br>KDLEEVKAKVQPYLDDFQKKWQEEMELYRQKVEPLRAELQEGARQKLHELQ<br>EKLSPLGEEMRDRARAHVDALRTHLAPYSDELRQRLAARLEALKENGGARLA<br>EYHAKATEHLSTLSEKAKPALEDLRQGLLPVLESFKVSFLSALEEYTKKLNTQG<br>TPVTQEFWDNLEKETEGLRQEMSKDLEEVKAKVQPYLDDFQKKWQEEMELY<br>RQKVEPLRAELQEGARQKLHELQEKLSPLGEEMRDRARAHVDALRTHLAPYS<br>DELRQRLAARLEALKENGGARLAEYHAKATEHLSTLSEKAKPALEDLRQGLLP<br>VLESFKVSFLSALEEYTKKLNTQGTPVTQEFWDNLEKETEGLRQEMSKDLEEV<br>KAKVQPYLDDFQKKWQEEMELYRQKVEPLRAELQEGARQKLHELQEKLSPL<br>GEEMRDRARAHVDALRTHLAPYSDELRQRLAARLEALKENGGARLAEYHAKA<br>TEHLSTLSEKAKPALEDLRQGLLPVLESFKVSFLSALEEYTKKLNTQGTPVTQE<br>FWDNLEKETEGLRQEMSKDLEEVKAKVQPYLDDFQKKWQEEMELYRQKVEP<br>LRAELQEGARQKLHELQEKLSPLGEEMRDRARAHVDALRTHLAPYSDELRQR<br>LAARLEALKENGGARLAEYHAKATEHLSTLSEKAKPALEDLRQGLLPVLESFK<br>VSFLSALEEYTKKLNTQLPGTGAAALESTFSKLREQLGPVTQEFWDNLEKETE<br>GLRQEMSKDLEEVKAKVQPYLDDFQKKWQEEMELYRQKVEPLRAELQEGAR<br>QKLHELQEKLSPLGEEMRDRARAHVDALRTHLAPYSDELRQRLAARLEALKE<br>NGGARLAEYHAKATEHLSTLSEKAKPALEDLRQGLLPVLESFKVSFLSALEEYT<br>KKLNTQGTPVTQEFWDNLEKETEGLRQEMSKDLEEVKAKVQPYLDDFQKKW<br>QEEMELYRQKVEPLRAELQEGARQKLHELQEKLSPLGEEMRDRARAHVDAL<br>RTHLAPYSDELRQRLAARLEALKENGGARLAEYHAKATEHLSTLSEKAKPALE<br>DLRQGLLPVLESFKVSFLSALEEYTKKLNTQGTPVTQEFWDNLEKETEGLRQE<br>MSKDLEEVKAKVQPYLDDFQKKWQEEMELYRQKVEPLRAELQEGARQKLHE<br>LQEKLSPLGEEMRDRARAHVDALRTHLAPYSDELRQRLAARLEALKENGGAR<br>LAEYHAKATEHLSTLSEKAKPALEDLRQGLLPVLESFKVSFLSALEEYTKKLNT<br>QLPGTGAAALEVPTIVMVDAYKRYK
spNW100	MGSSHHHHHHSSGLVPRGSHMASMTGGQQMGRGSGAMVTTLSGLSGEQG<br>PSGDMTTEEDSATHIKFSKRDEDGRELAGATMELRDSSGKTISTWISDGHVKD<br>FYLYPGKYTFVETAAPDGYEVATAITFTVNEQGQVTVNGEATKGDAHTSTFSK<br>LREQLGPVTQEFWDNLEKETEGLRQEMSKDLEEVKAKVQPYLDDFQKKWQE<br>EMELYRQKVEPLRAELQEGARQKLHELQEKLSPLGEEMRDRARAHVDALRTH<br>LAPYSDELRQRLAARLEALKENGGARLAEYHAKATEHLSTLSEKAKPALEDLR

	QGLLPVLESFKVSFLSALEEYTKKLNTQGTPVTQEFWDNLEKETEGLRQEMS<br>KDLEEVKAKVQPYLDDFQKKWQEEMELYRQKVEPLRAELQEGARQKLHELQ<br>EKLSPLGEEMRDRARAHVDALRTHLAPYSDELRQRLAARLEALKENGGARLA<br>EYHAKATEHLSTLSEKAKPALEDLRQGLLPVLESFKVSFLSALEEYTKKLNTQG<br>TPVTQEFWDNLEKETEGLRQEMSKDLEEVKAKVQPYLDDFQKKWQEEMELY<br>RQKVEPLRAELQEGARQKLHELQEKLSPLGEEMRDRARAHVDALRTHLAPYS<br>DELRQRLAARLEALKENGGARLAEYHAKATEHLSTLSEKAKPALEDLRQGLLP<br>VLESFKVSFLSALEEYTKKLNTQGTPVTQEFWDNLEKETEGLRQEMSKDLEEV<br>KAKVQPYLDDFQKKWQEEMELYRQKVEPLRAELQEGARQKLHELQEKLSPL<br>GEEMRDRARAHVDALRTHLAPYSDELRQRLAARLEALKENGGARLAEYHAKA<br>TEHLSTLSEKAKPALEDLRQGLLPVLESFKVSFLSALEEYTKKLNTQGTPVTQE<br>FWDNLEKETEGLRQEMSKDLEEVKAKVQPYLDDFQKKWQEEMELYRQKVEP<br>LRAELQEGARQKLHELQEKLSPLGEEMRDRARAHVDALRTHLAPYSDELRQR<br>LAARLEALKENGGARLAEYHAKATEHLSTLSEKAKPALEDLRQGLLPVLESFK<br>VSFLSALEEYTKKLNTQLPGTGAAALESTFSKLREQLGPVTQEFWDNLEKETE<br>GLRQEMSKDLEEVKAKVQPYLDDFQKKWQEEMELYRQKVEPLRAELQEGAR<br>QKLHELQEKLSPLGEEMRDRARAHVDALRTHLAPYSDELRQRLAARLEALKE<br>NGGARLAEYHAKATEHLSTLSEKAKPALEDLRQGLLPVLESFKVSFLSALEEYT<br>KKLNTQGTPVTQEFWDNLEKETEGLRQEMSKDLEEVKAKVQPYLDDFQKKW<br>QEEMELYRQKVEPLRAELQEGARQKLHELQEKLSPLGEEMRDRARAHVDAL<br>RTHLAPYSDELRQRLAARLEALKENGGARLAEYHAKATEHLSTLSEKAKPALE<br>DLRQGLLPVLESFKVSFLSALEEYTKKLNTQGTPVTQEFWDNLEKETEGLRQE<br>MSKDLEEVKAKVQPYLDDFQKKWQEEMELYRQKVEPLRAELQEGARQKLHE<br>LQEKLSPLGEEMRDRARAHVDALRTHLAPYSDELRQRLAARLEALKENGGAR<br>LAEYHAKATEHLSTLSEKAKPALEDLRQGLLPVLESFKVSFLSALEEYTKKLNT<br>QGTPVTQEFWDNLEKETEGLRQEMSKDLEEVKAKVQPYLDDFQKKWQEEME<br>LYRQKVEPLRAELQEGARQKLHELQEKLSPLGEEMRDRARAHVDALRTHLAP<br>YSDELRQRLAARLEALKENGGARLAEYHAKATEHLSTLSEKAKPALEDLRQGL<br>LPVLESFKVSFLSALEEYTKKLNTQGTPVTQEFWDNLEKETEGLRQEMSKDLE<br>EVKAKVQPYLDDFQKKWQEEMELYRQKVEPLRAELQEGARQKLHELQEKLS<br>PLGEEMRDRARAHVDALRTHLAPYSDELRQRLAARLEALKENGGARLAEYHA<br>KATEHLSTLSEKAKPALEDLRQGLLPVLESFKVSFLSALEEYTKKLNTQLPGTG<br>AAALEVPTIVMVDAYKRYK

SpyCatcher, orange; SpyTag, red.
