# Supplementary figures and images for: One-step construction of circularized nanodiscs using SpyCatcher-SpyTag
Source: Nat Commun. 2021 Sep 14;12:5451. doi: 10.1038/s41467-021-25737-7 (PMC8440770; doi:10.1038/s41467-021-25737-7)

Fig. 1b and Supplementary Fig. 1c

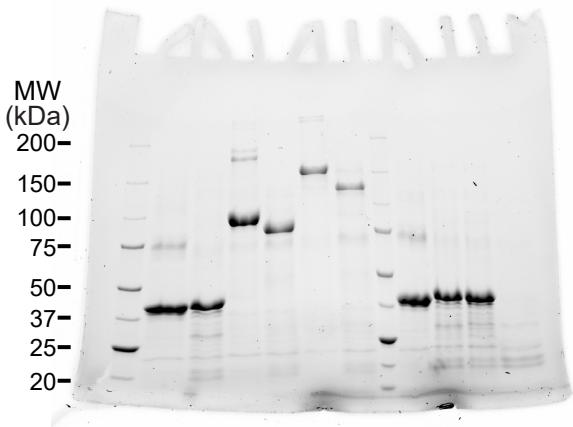

Fig. 4a

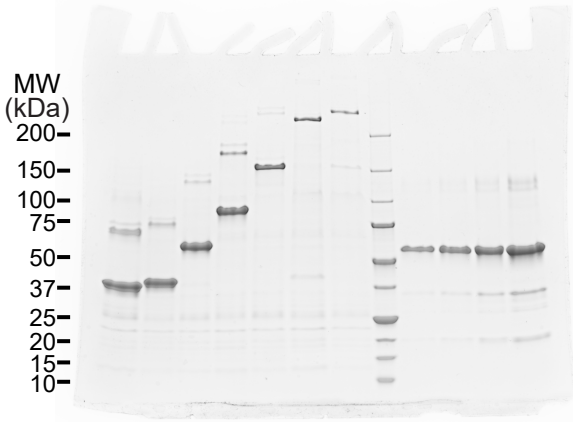

Supplementary Fig. 1a

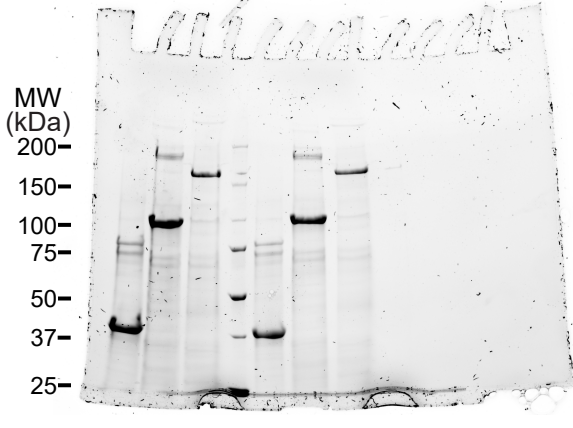

Supplementary Fig. 4a

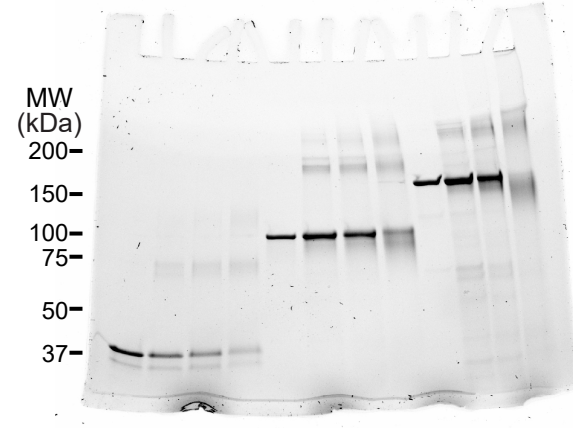

Supplement: Supplementary file 4 — Source Data [file 41467_2021_25737_MOESM4_ESM.zip › source data for gel images B.pdf]

**a**

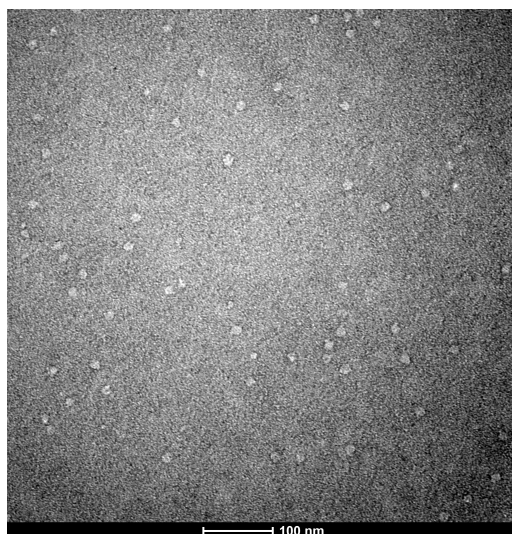

**b**

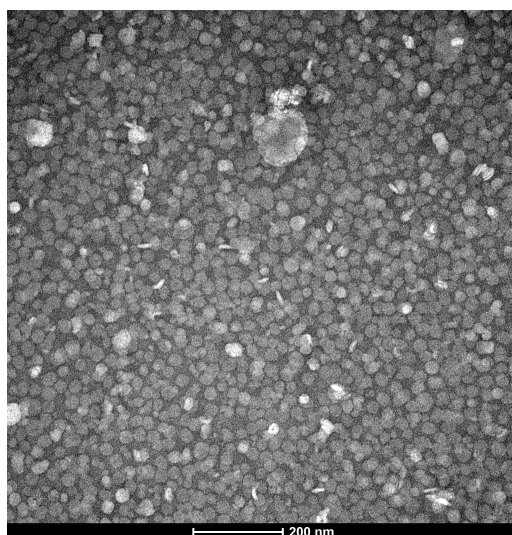

**c**

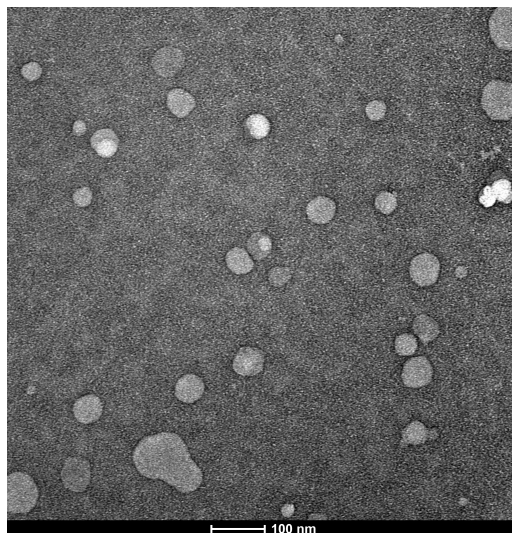

Supplement: Supplementary file 4 — Source Data [file 41467_2021_25737_MOESM4_ESM.zip › source EM micrographs for Figure 3.pdf]

**b**

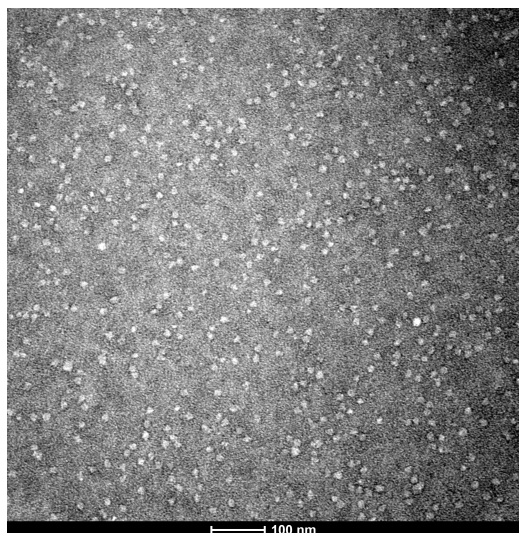

**c**

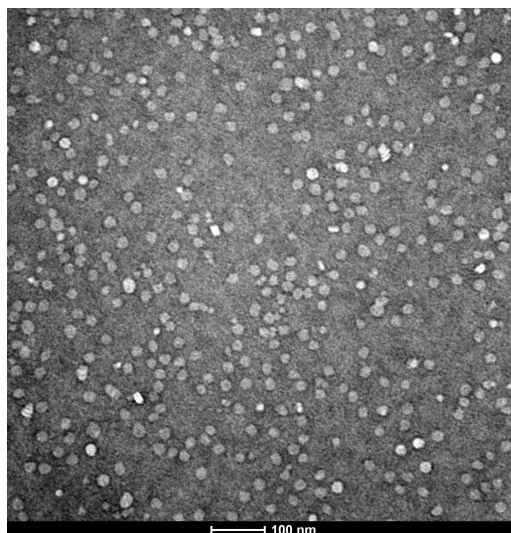

**d**

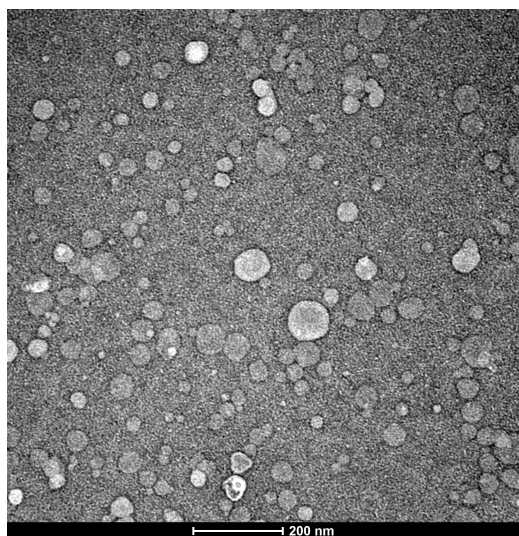

**e**

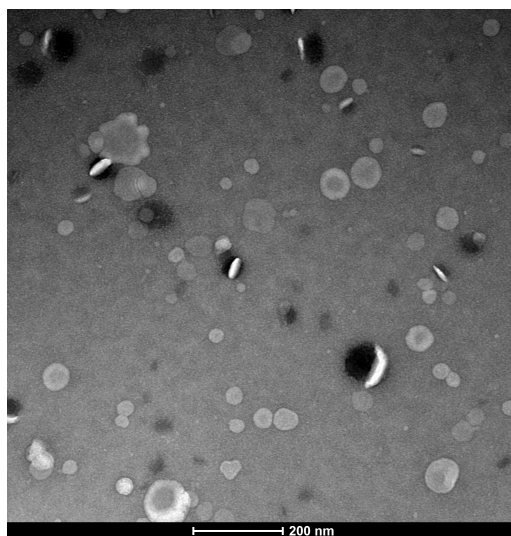

Supplement: Supplementary file 4 — Source Data [file 41467_2021_25737_MOESM4_ESM.zip › source EM micrographs for Figure 4.pdf]
